# Better Governance and Skilled Health Workforce Density: Are They Twin Catalysts for Health Outcomes in Sub‐Saharan Africa?

**DOI:** 10.1002/puh2.70235

**Published:** 2026-04-21

**Authors:** Michael Kouadio

**Affiliations:** ^1^ The Pan‐African University Institute of Governance, Humanities, and Social Sciences of Yaoundé Yaoundé Cameroon

**Keywords:** governance, health outcomes, skilled health workforce, sub‐Saharan Africa, two‐step generalised method of moments

## Abstract

Sub‐Saharan Africa (SSA) faces a persistent shortage of skilled health workers, which constrains health system performance and population health outcomes. Although previous studies examine either health workforce density or governance quality separately, little empirical evidence exists on their interaction. This study investigates the relationship between skilled health workforce density and health outcomes in 45 SSA countries over the period 1996–2023, with particular attention to the moderating role of governance quality. Using a dynamic panel approach and the two‐step system generalised method of moments (GMM) estimator, the analysis evaluates the impact of health workforce density on life expectancy (LE), maternal mortality and under‐5 mortality. The results show that greater availability of skilled health professionals significantly improves health outcomes, particularly by increasing LE and reducing maternal mortality. Furthermore, governance quality strengthens the effectiveness of health workforce investments, indicating that institutional capacity is crucial to translating human resources into better health outcomes. Control variables, such as GDP per capita, government health expenditure and primary education, also contribute to improved population health. The findings highlight the importance of integrating governance reforms with policies aimed at expanding the health workforce. Strengthening institutional quality alongside investments in medical training and workforce retention can provide a sustainable pathway to improving health outcomes in SSA.

## Graphical abstract

Stronger governance enhances the effectiveness of the skilled health workforce in Sub‐Saharan Africa. Increased health workforce density improves life expectancy and reduces under‐five and maternal mortality. Governance quality moderates this relationship by reducing corruption, strengthening accountability, and ensuring efficient resource allocation. Additionally, economic and structural factors, such as GDP per capita, government health expenditure, education, urbanisation, and access to safe water and sanitation, further shape health outcomes.

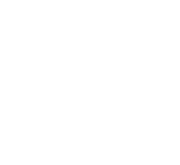

## 1 Introduction

Enhancing health outcomes is a core objective of national development agendas, aligning with the United Nations’ Sustainable Development Goal 3 [1] and the African Union's Agenda 2063 Goal 3 [2]. Recognising the critical role of health in driving economic growth, governments allocate substantial public resources to healthcare systems to improve population well‐being and overall economic performance [3]. Poor health diminishes individual well‐being, reduces labour productivity and threatens national economic stability. As a fundamental aspect of human capital, health has a direct impact on workforce performance by shaping both physical and cognitive abilities. Declining health reduces labour force participation and limits engagement in productive and social activities [4, 5, 6]. Moreover, poor health conditions degrade labour quality and hinder economic growth by slowing population development and lowering GDP per capita. Moreover, the World Health Organisation [7] highlights the strong correlation between persistent underdevelopment and poor population health. A healthy population is, therefore, a critical driver of economic development, as adequate health enables participation in both market and non‐market activities, facilitating the accumulation of human capital and long‐term economic growth. Achieving this requires sustained investment in healthcare financing, strategic resource allocation and the strengthening of health systems.

Despite efforts to enhance healthcare infrastructure, many African countries continue to face significant health challenges, including high disease burdens, inadequate medical facilities and disparities in healthcare access between rural and urban populations. These issues are compounded by insufficient healthcare funding, weak governance structures, and, in particular, shortages of skilled professionals, which limit the effective implementation of policies and service delivery [7]. Meanwhile, the health workforce remains the cornerstone of every health system, serving as a driving force to ensure that health activities are implemented smoothly for long‐term socioeconomic growth. Indeed, a well‐trained and appropriately distributed health workforce guarantees that health services are accessible, equitable and of high quality, thus boosting long‐term socioeconomic growth and enhancing the resilience of health systems [8].

Although evidence strongly links health workforce density to better health outcomes, the mechanisms and contextual factors shaping this relationship remain underexplored, inviting further investigation. In other words, health workers help save lives and promote health [9]. With an estimated 82,949 physicians, sub‐Saharan Africa (SSA) has an average of 12.5 physicians per 100,000 people, representing only 3% of the global healthcare workforce, a stark contrast to the global average of 152 per 100,000 [10]. This density remains well below the WHO benchmark of 44.5 skilled health workers per 10,000 population, the threshold required to achieve universal health coverage (UHC) [11]. Globally, there was a shortage of 7.2 million health workers, a figure projected to rise to 18 million by 2030 [12]. The study also revealed that 83 countries were below the WHO's minimum threshold of 22.8 health professionals per 10,000 population, a benchmark originally set to ensure the delivery of essential health services in line with Millennium Development Goals (MDGs) 4 and 5.

Life expectancy (LE) in most SSA countries averages around 59 years, considerably lower than the 80.3 years observed in OECD countries [13, 14]. Similarly, maternal health outcomes show stark disparities. Although the SDGs aim to reduce the global maternal mortality ratio (MMR) to fewer than 70 deaths per 100,000 live births by 2030, OECD countries had an MMR of 10.9 deaths per 100,000 live births in 2020, well below this target. In contrast, SSA recorded an MMR of 542 deaths per 100,000 live births in 2020, accounting for 69% of global maternal deaths [15]. These figures highlight the urgent need to prioritise maternal mortality interventions in SSA.

Infant mortality remains a critical challenge as well. In 2021, OECD countries had an average infant mortality rate of 4 deaths per 1000 live births [14], whereas globally, approximately 4.1 million infants died in the same year, representing 75% of all under‐5 deaths. In SSA, infant mortality stood at 52.7 per 1000 live births in 2018, far above the SDG target of 25 per 1000 by 2030 [16].

These disparities are not merely demographic or economic concerns; they reflect broader issues in human capital development. SSA consistently ranks among the regions with the lowest health‐human capital indicators [17], underscoring the need for targeted health interventions.

The acute shortage of physicians in SSA significantly impedes the delivery of essential health services, exacerbates systemic health disparities and hinders progress toward UHC. Addressing this challenge necessitates integrated policy interventions aimed at strengthening medical education, improving workforce retention and ensuring equitable distribution of healthcare professionals across urban and rural areas. Increasing the number of healthcare professionals, particularly physicians, is vital for bridging the gap between clinical service delivery and broader health system interventions. Beyond providing direct patient care, physicians contribute to improved health outcomes through leadership in public health initiatives, advocacy for evidence‐based policies and research that informs strategic health planning [18]. In addition to these responsibilities, physicians contribute to capacity‐building efforts by training other healthcare workers, strengthening community‐based health systems and implementing innovative service delivery models [19].

In SSA, for example, partnerships between healthcare professionals and local organisations have resulted in substantial improvements in maternal and child health outcomes through better trained health workers and increased community mobilisation [20]. Physicians also contribute to broader socio‐economic development by improving health outcomes through early diagnosis, preventive care and evidence‐based treatment. By reducing the disease burden, lowering mortality rates and increasing workforce productivity, they have a direct impact on overall development [20].

As health systems continue to face challenges related to physician shortages, it is clear that a well‐trained and adequately supported physician workforce is essential for effective healthcare delivery. Shortages of skilled professionals frequently result in service inefficiencies, delayed treatments and poorer patient outcomes [21]. Strengthening healthcare workforce policies, investing in physician education and implementing strategies for fair compensation and retention are critical to fostering sustainable healthcare systems, particularly in African countries [9]. Addressing physician shortages through strategic workforce planning, international collaboration and local innovations is crucial to enhancing the resilience and effectiveness of the healthcare system. Ensuring that populations have access to timely and quality medical care depends on these initiatives. In parallel, integrating governance reforms into health sector management can further improve service delivery and policy effectiveness.

Strong governance systems are essential for building resilient healthcare infrastructures capable of addressing evolving health challenges [22]. Such systems promote transparency, inclusion and efficiency in resource allocation, ensuring that healthcare services reach those most in need [8]. By enhancing institutional capacity and streamlining policy coordination, governance frameworks enable the development and implementation of responsive health policies that address both immediate and long‐term public health concerns [23].

Governance structures that prioritise equity reduce healthcare disparities and strengthen system resilience. Accountability mechanisms within these frameworks prevent corruption and inefficiency, ensuring that healthcare funds are allocated effectively to improve service delivery and infrastructure. During crises, such as pandemics or natural disasters, strong governance allows health institutions to respond rapidly, reducing risks and safeguarding public well‐being. Participatory and data‐driven governance further supports evidence‐based decision‐making, resulting in sustainable health policies and improved population health outcomes [24].

Government efficiency also directly affects health outcomes. Inefficient governance can hinder resource allocation and public health policy implementation, limiting improvements in health outcomes [25, 26]. Conversely, efficient governance fosters innovation in healthcare, such as the development of advanced medications and medical equipment, thereby enhancing treatment effectiveness [27]. Furthermore, efficient governments are better positioned to enforce anti‐corruption regulations, promoting transparency, accountability and more effective allocation of medical resources [28, 29, 30]. Together, these mechanisms demonstrate how strong and efficient governance is critical for achieving better health outcomes.

Despite extensive research on health outcomes, most studies examine health workforce density and governance quality separately, with limited attention to their combined effects. Although greater health workforce availability is widely acknowledged as improving health outcomes, this relationship often presupposes functional and efficient institutions. In SSA, many health systems are constrained by governance challenges, including weak institutional capacity, corruption and ineffective policy implementation. These persistent governance deficiencies may significantly attenuate the benefits of health workforce investments, yet empirical evidence on this interaction in SSA remains scarce. Given the region's unique institutional, social and economic contexts characterised by high disease burdens, resource limitations and complex policy environments, understanding how governance moderates health workforce effectiveness is critical for informing evidence‐based interventions and policy reforms. Addressing this knowledge gap will provide insights into optimising health system performance and improving population health in SSA [23, 26, 30, 31]. Furthermore, much of the existing evidence comes from high‐ or middle‐income countries, with limited focus on SSA, where health workforce shortages and governance challenges interact to exacerbate poor health outcomes, such as high maternal and under‐5 mortality rates and low LE [32, 33]. This study addresses existing gaps by simultaneously examining health workforce density and governance quality and their combined impact on health outcomes in SSA. Using panel data from 1996 to 2023 and grounded in institutional theory and Grossman's health production framework, the analysis investigates how governance moderates the relationship between health workforce density and health outcomes. The study employs the two‐step system generalised method of moments (GMM) estimator to account for endogeneity, omitted variable bias and unobserved heterogeneity.

By adopting this integrated approach, the study contributes context‐specific empirical evidence to an underexplored area of global health research and provides actionable insights for health sector policy. It posits that merely increasing the supply of health professionals is insufficient without governance reforms that promote transparent resource allocation, curb corruption and strengthen accountability. Ultimately, this framework aims to inform more comprehensive and sustainable strategies for improving health outcomes across SSA.

The study is structured as follows: After the introduction (Section 1), the review of the existing literature on the relationship between the health workforce density and health outcomes as well as the moderating role of governance quality on health workforce density and health outcomes (Section 2) present the methods, and data in Sections 3 and 4 present the main results and the discussion. Finally, Section 5 draws the conclusion and policy recommendations.

## 2 Literature Review

This study is theoretically grounded in institutional theory [34], which posits that the performance and outcomes of organisations and systems, such as health systems, are shaped by the formal and informal rules, norms and governance structures within which they operate. Governance quality, including indicators, such as corruption control and government effectiveness, represents institutional conditions that determine how health resources are allocated, managed and translated into outcomes. This framework explains how weak institutions can distort the expected benefits of increasing physician supply by enabling inefficiencies, corruption and a lack of accountability. The study is also based on Grossman's theory [35, 36], which offers a suitable theoretical foundation for analysing health demand and presents a coherent framework for examining optimal health trajectories and longevity [36]. A key benefit of estimating an aggregate health production function is that it enables the assessment of the impact of the role of governance quality and the physicians’ relationship on health outcomes.

Empirically, understanding the determinants of health outcomes requires examining not only the availability of healthcare providers but also the quality of patient–clinician interactions. Although geographic disparities in the distribution of health professionals have been associated with variations in healthcare outcomes, the specific relationship between primary care physician (PCP) density and health outcomes remains insufficiently explored. Anyangwe and Mtonga [9] examined inequities in the global health workforce and identified them as a major barrier to health in SSA countries. The study revealed that SSA countries need to increase their health workforce by approximately 140% to ensure adequate coverage of essential health services capable of improving health outcomes, such as LE. Thus, there is an urgent need to address the global health workforce crisis, which requires shared global responsibility, strong political will, and sustained financial investment.

In the same vein, in a US context, Jabbar et al. [32] used county‐level data from 2019 to show a strong negative correlation between PCP density and mortality from all causes and cardiovascular disease, even after adjusting for the Social Vulnerability Index. Although their study suggests that increased PCP access improves population health, it does not investigate the mechanisms through which this occurs or the role of governance quality, nor does it extend findings to low‐ and middle‐income countries (LMICs).

Rosser et al. [37] make use of Demographic and Health Survey (DHS) data from 35 SSA countries from 2008 to 2017 to investigate the effect of healthcare worker density on maternal health service utilisation (facility‐based delivery and antenatal care uptake). The researchers discovered that each additional physician per 1000 persons increased the risk of facility birth by 9.8%, whereas each additional nurse or midwife increased it by 8.9%. To enhance maternal health outcomes, the study suggests investing in the health workforce, particularly by increasing the number of mid‐level workers. However, the study failed to consider the significance of governance in the analysis, which could function as a lubricant in the health workforce and health outcomes.

Nguyen et al. [38] employed panel regression analyses using provincial‐level data from 63 provinces in Vietnam between 2006 and 2013 to assess the influence of the health workforce on health outcomes. Their study demonstrated that an increase of one physician per 10, 000 population was associated with an approximate 4.1‐month rise in LE. Furthermore, the presence of midwives and pharmacists exerted a more pronounced effect on reducing neonatal and under‐5 mortality rates. On the basis of these findings, the authors underscored the importance of pursuing balanced investments across all health professional cadres, rather than concentrating exclusively on physicians. However, the study did not explicitly take governance‐related issues into consideration, such as corruption, regulatory quality, accountability and institutional capacity, which could influence the association between workforce availability and health outcomes. These governance elements could have a considerable impact on how well health workers are deployed, managed and maintained within the system.

Similarly, Liang et al. [39] examined the relationship between health workforce density and under‐5 mortality in rural China, utilising longitudinal county‐level data from 2871 counties between 2003 and 2011. Their analysis revealed that a 1% increase in the density of health workers was associated with a 0.133% reduction in under‐5 mortality, with the impact being more substantial in less‐developed areas. To improve child health outcomes, the authors recommended expanding the health workforce and promoting a more equitable distribution of health professionals across rural regions. However, the study did not consider the potential role of governance quality in shaping health service delivery, an increasingly recognised determinant of health system performance.

Several studies have documented the influence of governance on health outcomes. Ref. [40] shows that effective governance is essential for realising health improvements in countries with decentralised fiscal systems. Ref. [41] also provides a comprehensive review highlighting the strong association between governance quality and population health. Building on this literature, the present study investigates how governance quality interacts with skilled health workforce density to affect LE, under‐5 mortality and maternal mortality in SSA, providing novel insights on governance as a moderator rather than merely a main effect.

Despite the critical importance of governance quality in shaping this relationship, few studies investigate key governance indicators, such as government effectiveness [42, 43]. This interaction is particularly relevant in SSA, where clinician shortages, unequal distribution and governance challenges affect both access and quality of care. For instance, Kelley et al. [44] conducted a systematic review and meta‐analysis of 13 randomised controlled trials (RCTs) in Europe, the US and Australia, finding a modest yet significant positive effect of improved patient–clinician relationships on health outcomes. However, the study did not examine how clinician availability or institutional quality might influence the effectiveness of these relationships, especially in contexts like SSA.

Thomas et al. [33] assessed Brazil's Programa Mais Médicos using a generalised synthetic control estimator and found no significant impact on hospitalisation or mortality rates. Their analysis focused on statistical associations and overlooked contextual mediators, such as governance quality, which may affect how additional physicians influence health outcomes. This gap is particularly salient for LMICs, where institutional inefficiencies can undermine healthcare interventions.

Corruption is widely recognised as a barrier to effective health systems, especially in LMICs. Gupta et al. [45] and Pandolfelli et al. [46] emphasise that corruption undermines health system efficiency and equity by misallocating resources and compromising service delivery. It also contributes to the presence of ghost workers, absenteeism and reduced morale among health professionals, ultimately lowering health service availability and quality. However, existing research rarely examines how corruption interacts with skilled health workforce variables like physician density or broader governance indicators like government effectiveness.

Gaitonde et al. [47] argue that systemic corruption undermines health system foundations, whereas Nadpara and Samanta [31] find its effects more severe in poorer countries. Yet, these studies do not explore how skilled health density availability might moderate these effects or the specific challenges in SSA. Similarly, Saltman et al. [48] stress that corruption in public health financing leads to resource leakage and reduced service provision, especially for vulnerable populations. Nevertheless, empirical studies linking specific governance indicators (e.g., corruption control and government effectiveness) with health outcomes across countries remain limited. This gap impedes a full understanding of how better governance could enhance primary healthcare systems and reduce inequities, particularly in LMICs. Moreover, Achim et al. [49] show that corruption negatively impacts physical and mental health, with stronger effects in economically disadvantaged countries. However, they do not account for skilled health workforce capacity, such as physician density, as a potential moderator. Văidean et al. (2021) similarly find that corruption worsens child mortality and LE across the EU but neglect the role of workforce distribution.

In the West African context, [50] find that stronger anti‐corruption measures are linked to improved health outcomes in 11 countries. However, their study omits other governance aspects like government effectiveness and workforce‐related variables, both crucial to service delivery. Ding et al. [51] demonstrate that government effectiveness significantly improves health outcomes using global panel data. Still, their analysis lacks regional specificity and overlooks interactions with skilled health workforce dynamics.

In summary, although existing studies highlight the importance of skilled health workforce density and the detrimental effects of corruption on health systems, few investigate how governance quality and workforce capacity interact to influence health outcomes, particularly in SSA. Research often treats these variables in isolation, neglecting how weak governance may limit the benefits of increasing physician supply through inefficiencies and mismanagement. Institutional factors are also seldom considered in studies of patient–clinician dynamics. This highlights a critical gap: the need for integrated, context‐specific research that examines how physician density and governance quality jointly influence health outcomes, particularly in regions with fragile health systems.

In summary, although existing research emphasises the significance of skilled health workforce density and the detrimental effects of corruption on health systems, few studies have examined the role of governance quality and skilled health workforce capability in influencing health outcomes, particularly in SSA. Most evaluations handle these variables separately, failing to consider how weak governance systems might impair the potential benefits of greater physician supply through inefficiencies, resource misallocation and poor accountability. Furthermore, governance dimensions are infrequently considered in studies of patient–clinician interactions, despite their importance to service quality and healthcare system performance. This identifies a significant gap in the literature: the need for integrated, context‐sensitive research on how physician density and governance quality interact to influence health outcomes, particularly in areas with unstable institutional settings.

## 3 Methodology

To examine the relationship between the skilled health workforce and health outcomes in SSA, this study is grounded in the health production function framework developed by Grossman [35]. In its original formulation, the model conceptualises social, economic and environmental factors as inputs into the health production system; in this study, governance quality is incorporated as an additional determinant. Grossman's [35, 36] health production function can be expressed as follows:

(1) $$H=F\left(X\right)$$

where *H* denotes a measure of an individual's health, and *X* represents a vector of explanatory variables influencing health status (LE at birth, under‐5 mortality rate and maternal mortality), which include GDP per capita, people using safely managed drinking water services (% of population), people using at least basic sanitation services (% of population), rural population (% of total population), school enrolment, primary (% gross), domestic general government health expenditure (% of GDP) and access to electricity. They are commonly used to explain health outcomes [52].

Higher GDP per capita improves health by increasing access to medical services, better nutrition and healthier living conditions [43]. Likewise, health expenditure, both public and private, enhances healthcare delivery and infrastructure, ensuring the availability of essential medicines and reducing mortality [26]. Urbanisation also affects health outcomes by improving access to facilities and information; however, if poorly managed, it can lead to overcrowding, poor sanitation and environmental problems that harm health [52]. Education levels, particularly female education, are consistently linked to better health outcomes through improved health knowledge, health‐seeking behaviour and empowerment to access and utilise healthcare services [43, 52]. Access to improved water sources is essential for public health, as it helps prevent waterborne diseases, promotes hygiene and reduces child mortality, particularly in low‐resource settings [8, 52].

Additionally, limited access to clean water is often linked to higher rates of communicable diseases. Similarly, access to electricity supports better health outcomes by enabling essential services in health facilities, such as lighting, refrigeration for vaccines and the use of medical equipment [26]; Adair‐Rohani et al. [53]. Moreover, electricity facilitates health education through communication technologies and contributes to broader development processes that enhance population well‐being.

The estimation of the health production function highlighted in Equation (2) requires comprehensive data encompassing health outcomes and governance quality, as well as socio‐economic variables. Within this study, LE at birth, infant mortality rate and MMR are employed as the principal health outcome measures. These indicators are extensively utilised in the literature as a robust and interpretable proxy for mortality rates [17, 26, 45, 52].

The main explanatory variables of interest consist of the skilled health workforce (physicians per 1000 people and nurses and midwives per 1000 people). The skilled health workforce density variable is constructed as the combined density of physicians, nurses and midwives per 1000 population, consistent with the definition of skilled health personnel provided by the World Health Organisation. This composite measure captures the overall availability of clinically trained professionals involved in service delivery [7].

Governance Quality: Governance data are sourced from the World Bank's Worldwide Governance Indicators (WGI) database. Following established practice [54], six governance dimensions are considered: government effectiveness, control of corruption, regulatory quality, voice and accountability, rule of law and political stability and absence of violence. To obtain a single summary measure, the unweighted average of these six indicators for each country‐year is computed. This aggregate value ranges from approximately −2.5 (weak governance) to 2.5 (strong governance), with higher values indicating better governance performance. Missing values for any indicator were replaced with the country's average for that dimension across available years; if any indicator remained missing after this imputation, the corresponding country‐year observation was excluded from the main analysis.

The unweighted average is used because the six governance dimensions are strongly correlated, and the simple average provides a transparent, interpretable measure of overall governance quality. Although methods, such as principal component analysis (PCA), could be used to create a composite index, the simple average is widely adopted in cross‐country empirical research [54] and avoids the potential arbitrariness of weighting schemes, ensuring that each governance dimension contributes equally to the summary measure.

The interaction term (skilled health workforce and the aggregate value of governance) reflects how governance quality moderates the impact of the skilled health workforce on health outcomes. Although inadequate governance hinders the delivery of public services, particularly in the health sector, robust and effective institutions can alleviate these challenges. In environments characterised by good governance capacity demonstrated through quality public services, an autonomous and skilled civil service and effective policy implementation, health systems are better positioned to efficiently deploy resources, leading to enhanced health outcomes [55].

(2) $$\begin{array}{ccc} {\ln\;\mathrm{Ho}}_{it} & = & \beta_{0}+\beta_{1}{\ln\;\mathrm{Ho}}_{it-1}\beta_{2}\;\mathrm{Skilled}\;\mathrm{Health}\_{\mathrm{Workforce}}_{it}\\ & & +\;\beta_{3}\mathrm{GO}V_{it}+\beta_{4}\mathrm{GOV}\times \mathrm{Skilled}\_\mathrm{Health}\_\mathrm{Workforc}e_{it}\\ & & +\;\beta_{3}\ln\;X_{it}+\mu_{i}+\omega_{t}+\varepsilon_{it} \end{array}$$

$\mathrm{where}\;Ho_{it}$ is the health outcomes: LE at birth, under‐5 mortality rate and maternal mortality, country *i*, time *t*. Skilled_Health_Workforce, the skilled health workforce density variable, is constructed as the combined density of physicians, nurses and midwives per 1000 population. $\mathrm{GOV}\times \mathrm{Skilled}\;\mathrm{Health}\_\mathrm{Workforc}e_{it}$ is the interaction term between government value and skilled health workforce to test conditional effects. $X_{it}$ is the control variables. $\mu_{i}$ is the unobserved country‐specific effect. $\omega_{t}$ is the time‐specific effect. $\varepsilon_{it}$ is the error term.

This study employs a quantitative research design based on panel data to assess the joint effects of physician density and governance quality on health outcomes across SSA countries. The empirical analysis spans the period 1996–2023, subject to data availability, and utilises a balanced panel comprising countries for which complete and relevant data are accessible. Missing observations are addressed using the median imputation method, following the approach outlined by Little and Rubin [56], which is appropriate given the relatively low proportion of missing data and the robustness of the median to outliers. The skilled health workforce density variable is constructed as the combined density of physicians, nurses and midwives per 1000 population, consistent with the WHO definition of skilled health personnel, and data are drawn from the World Bank's World Development Indicators (WDI).

In this panel dataset spanning 1996–2023, missing values are minimal, less affecting the observations for key variables (skilled health workforce density, governance indicators, GDP per capita and health outcomes). To maintain consistency and avoid bias from listwise deletion, median imputation was applied to replace missing values. This approach is appropriate given the small proportion of missing data and the robustness of the median to outliers, ensuring that the central tendency of each variable is preserved without artificially inflating variability [56]. Observations with missing values for more than one key variable were excluded from the main analysis to maintain data quality.

To address the potential endogeneity of explanatory variables, along with country‐specific effects and omitted variable bias, this study employs the two‐step GMM estimator with lagged one level of the dependent variable as instruments developed by Arellano and Bond [57]. Endogeneity arises when explanatory variables are correlated with the error term, leading to biased estimates. This method uses lagged levels of the endogenous variables as instruments to mitigate this issue effectively. Although the traditional two‐stage least squares (2SLS) estimator can handle some endogeneity problems, system GMM offers a more efficient approach when multiple endogenous variables are present by combining equations in differences with equations in levels, thereby exploiting additional moment conditions. This method controls not only for endogeneity but also for unobserved panel heterogeneity, autocorrelation, measurement errors and omitted variable bias, as highlighted by Ullah et al. [58]. Unlike Difference GMM, which removes fixed effects by first differencing and consequently subtracts the previous observation from the current one, thereby amplifying data loss, system GMM subtracts the average of all future available observations, preserving more information and improving estimation precision [59]. Furthermore, Bond [60] notes that Difference GMM may be biased in the presence of unit roots, whereas system GMM tends to produce more reliable and consistent estimates. Diagnostic tests in this study confirmed the presence of endogeneity, which makes System GMM particularly appropriate. The two‐step estimator further improves efficiency by producing robust standard errors that account for heteroscedasticity and autocorrelation. Given these advantages, the two‐step system GMM estimator is well‐suited to capture the dynamic relationships in the panel data and to address the econometric challenges inherent in the model, ensuring that the results are consistent, efficient and reliable.

## 4 Results and Discussions

Over the period 1996–2023, in Table 1, the average LE at birth in SSA is estimated at around 58.33 years, which is below 60 years. In our sample, the mean LE is 58.09 years, consistent with this regional estimate. The majority of countries in the region have life expectancies below 60 years, reflecting persistent health challenges. The wide range observed in the data, from as low as approximately 15 years to nearly 77 years, highlights substantial disparities in health outcomes across the region.

**TABLE 1:** Descriptive statistics.

Variable	Obs	Mean	Std. dev.	Minimum	Maximum
Life expectancy at birth	1260	58.091	7.432	14.665	77.237
Infant mortality rate	1260	60.314	27.337	9.4	234.9
Maternal mortality ratio	1260	512.717	414.341	37	7514
Physicians per 1000 people	1260	0.211	0.333	0.001	3.819
Nurses and midwives (per 1000 people)	1260	1.134	1.32	0.002	10.76
Governance aggregate value	1260	−0.659	0.535	−2.111	0.897
GDP per capita	1260	1963.102	2823.387	71.407	19, 141.512
Urban population (% of total population)	1260	39.118	16.87	7.412	91.029
People using at least basic sanitation services (% of population)	1260	32.348	22.892	0.04	100.604
People using at least basic drinking water services (% of population)	1260	62.28	17.197	12.872	100.081
Current health expenditure (% of GDP)	1260	5.683	4.381	0.639	47.904
Primary completion rate	1260	64.835	23.806	5.6	97.167

*Source:* Author from the WDI database (1996–2023).

Maternal and infant mortality rates remain high across SSA, reflecting significant health system challenges. In our sample, the average infant mortality rate is approximately 60 deaths per 1000 live births, with substantial variation across countries. Similarly, the MMR averages around 513 deaths per 100, 000 live births but shows wide dispersion, indicating that some countries face extreme maternal health risks. These high mortality figures highlight persistent gaps in access to quality maternal and child healthcare services throughout the region (Table 1).

Descriptive analysis of institutional quality indicates that it remains generally poor across SSA. In our study, the aggregated governance value shows a negative mean of −0.659, echoing previous results where all institutional dimensions of government effectiveness, control of corruption, regulatory quality, voice and accountability, rule of law, political stability and the absence of violence are negative.

Regarding GDP per capita, in our sample, the average GDP per capita is lower at $1963.10 but shows wide variation, from $71.41 to $19, 141.51, illustrating the economic diversity of the countries studied (Table 1).

Access to basic services is mixed. The average population with access to at least basic drinking water services is around 62.28%, slightly higher than the previously reported 60.98%. However, access to at least basic sanitation services is much lower, with a mean of only 32.35%, indicating significant challenges in sanitation. The urban population represents, on average, 39.12% of the total population, which aligns closely with the regional average rural population of about 38.38%, underlining the predominance of rural areas in the region (Table 1).

Human resources for health are scarce in our sample. There are, on average, 0.211 physicians and 1.134 nurses and midwives per 1000 people, highlighting limited access to health professionals in most countries, although some countries show much higher maximum values.

Finally, some variables require careful attention. The current health expenditure as a percentage of GDP shows an average of 5.68%, with values ranging from approximately 0.64% to 47.9%. This indicates considerable variation in the share of national resources devoted to health across countries in the sample. Similarly, the primary completion rate averages 64.84%, with a standard deviation of 23.81%, indicating moderate variability across observations (Table 1).

## 5 Descriptive Statistics

### 5.1 Findings Presentation

The findings in Tables 2, 3, 4 demonstrate that, at one point, the skilled health workforce coefficient improves health outcomes in SSA. LE rises by 0.034 years, whereas the MMR decreases by 0.044 points, and under‐5 mortality shows a negative association for every 1% increase in the skilled health workforce. The interaction term between governance aggregate value and health workforce density is positive and statistically significant (*β* = 0.399), indicating a moderating role of governance. GDP per capita and primary school enrolment are positively associated with LE, whereas urban population and government health expenditure (% of GDP) show mixed significance. Access to safe drinking water and sanitation has variable effects across the outcomes. Full regression results are presented in Tables 2, 3, 4.

**TABLE 2:** Relationship between skilled health workforce, governance quality and life expectancy at birth in sub‐Saharan Africa (SSA).

		Life expectancy at birth				
	Coeff.	St. err.	*t* value	*p* value	[95% Confidence interval]	Sig
Life expectancy at birth (Lag 1)	0.413	0.017	3.65	0.000	0.701–0.726	^***^
Health workforce	0.034	0.214	4.36	0.000	1.215–1.356	^***^
Governance aggregate value × skilled health workforce density	0.399	0.243	2.88	0.004	0.223–1.175	^***^
Governance aggregate value	0.134	0.117	6.29	0.057	0.363–0.505	^***^
Urban population	0.028	0.004	6.55	0.023	0.020–0.036	^***^
Sanitation	0.007	0.005	1.25	0.210	−0.004 to 0.017	
Access to basic drinking water services	0.002	0.007	0.23	0.820	−0.012 to 0.015	
Ln GDP per capita	0.129	0.048	2.67	0.007	0.035–0.224	^***^
Government health expenditure (%GDP)	0.012	0.003	0.13	0.898	−0.006 to 0.007	
Primary school enrolment	0.007	0.001	6.88	0.000	0.005–0.010	^***^
Constant	10.423	0.451	23.12	0.000	9.539–11.306	^***^
Mean dependent var.		58.302	SD dependent var.		7.356	
Number of observations		1215	Chi‐square		2103.048	
Number of groups		45				
Number of instruments		16				
*F*‐test of joint significance		(0.0000)				
AR (1)		0.000				
AR (2)		0.321				
Sargan test		0.000				
Hansen test		0.899				

* *p* < 0.1.

** *p* < 0.05.

*** *p* < 0.01.

*Source:* Author's compilation, WDI data (1996–2023).

**TABLE 3:** Relationship between health workforce, governance quality and under‐5 mortality rate in sub‐Saharan Africa (SSA).

Under‐5 mortality rate						
	Coeff.	St. err.	*t* value	*p* value	[95% Confidence interval]	Sig
Under‐5 mortality rate (Lag 1)	0.255	0.007	0.50	0.000	0.642–0.667	^***^
Skilled health workforce density	−0.475	1.123	−0.42	0.673	−2.677 to 1.727	
Governance aggregate value × skilled health workforce density	−0.320	0.816	−0.39	0.695	−1.920 to 1.279	
Governance aggregate value	−0.996	0.741	−1.35	0.179	−0.455 to 2.448	
Urban population	−0.086	0.015	−5.64	0.000	−0.117 to −0.056	^***^
Sanitation	−0.023	0.014	−1.63	0.104	−0.050 to 0.005	
Access to basic drinking water services	−0.027	0.018	−1.55	0.121	−0.007 to 0.061	
Ln GDP per capita	−0.346	0.259	−5.20	0.000	−1.853 to −0.839	^***^
Government health expenditure (%GDP)	−0.038	0.014	−2.73	0.006	−0.066 to −0.011	^***^
Primary school enrolment	−0.023	0.002	−9.30	0.000	−0.028 to −0.018	^***^
Constant	0.921	2.898	17.57	0.000	45.242–56.6	^***^
Mean dependent var.		59.330	SD dependent var.		26.704	
Number of observations		1215	Chi‐square		2559.166	
Number of instruments		18				
*F*‐test of joint significance		(0.0000)				
AR (1)		0.013				
AR (2)		0.521				
Sargan test		0.023				
Hansen test		0.427				

* *p* < 0.1.

** *p* < 0.05.

*** *p* < 0.01.

*Source:* Author's compilation, WDI data (1996–2023).

**TABLE 4:** Relationship between skilled health workforce, governance quality and maternal mortality ratio in sub‐Saharan Africa (SSA).

Maternal mortality rate						
ln Maternal	Coeff.	St. err.	*t* value	*p* value	[95% Confidence interval]	Sig
Maternal mortality rate (Lag 1)	0.493	0.010	2.50	0.000	0.874–0.911	^***^
Skilled health workforce	−0.044	0.021	−2.10	0.035	−0.085 to −0.003	^**^
Governance aggregate value × health workforce	−0.042	0.027	−1.55	0.121	−0.011 to 0.095	
Governance aggregate value	−0.104	0.008	−0.43	0.664	−0.012 to 0.022	
Urban population	−0.021	0.060	−1.12	0.263	−0.021 to 0.075	
Sanitation	−0.019	0.045	−3.77	0.001	−0.011 to −0.009	^***^
Access to basic drinking water services	−0.019	0.013	−0.82	0.412	0.437–0.874	
Ln GDP per capita	−0.010	0.023	−3.07	0.002	−0.017 to −0.004	^***^
Government health expenditure (%GDP)	−0.001	0.004	−3.53	0.035	−0.009 to −0.001	^***^
Primary school enrolment	−0.001	0.012	−5.74	0.035	−0.053 to −0.041	^***^
Constant	9.852	0.079	10.79	0.000	0.697–1.007	^***^
Mean dependent var.		5.955	SD dependent var.		0.796	
Number of observations		1215	Chi‐square		1864.701	
Number of instruments		11				
*F*‐test of joint significance		(0.0000)				
AR (1)		0.032				
AR (2)		0.412				
Sargan test		0.041				
Hansen test		0.127				

* *p* < 0.1.

** *p* < 0.05.

*** *p* < 0.01.

*Source:* Author's compilation, WDI data (1996–2023).

These results are consistent with the studies by Nguyen et al. [38], Liang et al. [39] and Liu and Eggleston [61], who found that greater availability of health workers, especially the overall density of skilled professionals and that of nursing and midwifery personnel, showed a significant association with improved health outcomes, notably lower maternal, under‐5, infant and neonatal mortality rates. Our results imply that strengthening the health workforce, adding to equitable distribution, is critical for improving population health in SSA countries.

Our findings further suggest that governance plays a crucial moderating role in the relationship between health professionals and health outcomes in SSA. Specifically, improvements in governance quality not only strengthen the effectiveness of the health workforce but also contribute directly to enhanced population health outcomes (Tables 2, 3, 4). These results are consistent with the work of Văidean et al. (2021), who found that corruption exacerbates child mortality and reduces LE across the European Union. Similarly, Ding et al. [51] demonstrated that government effectiveness significantly improves health outcomes, underscoring the importance of strong institutional capacity. In the African context, Anyangwe and Mtonga [9] highlighted that governance‐related constraints, such as corruption, inadequate planning and weak institutional frameworks, are major drivers of persistent health workforce shortages, which in turn undermine efforts to train, recruit and retain health professionals. In addition, Kruk et al. [62] showed that trust in government and accountability are critical determinants of citizens’ willingness to seek and utilise health services. Taken together, these studies reinforce the argument that improved governance is pivotal not only for expanding and sustaining the health workforce but also for fostering greater health service utilisation and, ultimately, better health outcomes.

The interaction between the governance aggregate value and skilled health workforce density in Table 2 is positive and statistically significant (*β* = 0.399), highlighting governance as a key moderator of health workforce effectiveness. This implies that a one‐point increase in the interaction term raises LE by 0.399 years in SSA. At the mean governance level, an additional skilled health worker per 1000 population has a moderate effect on LE, whereas at one standard deviation above the mean, the impact is substantially larger, indicating that stronger governance enhances the returns on health workforce investments. Conversely, in countries with weaker governance, the effect of additional health workers is limited. These results underline that governance quality is critical for translating health workforce density into tangible population health gains, consistent with evidence from [63].

These results suggest that merely expanding the number of doctors, nurses and midwives is insufficient to guarantee improved health outcomes unless underlying governance challenges, such as corruption, weak accountability and inadequate planning, are effectively addressed. Strong governance plays a critical role in ensuring that health resources are allocated transparently, salaries are delivered promptly and training programs are efficiently managed. By fostering accountability and reducing inefficiencies, good governance maximises the impact of health sector investments, minimises resource leakage and enhances the sustainability of workforce‐related interventions.

As presented in Tables 2, 3, 4, economic factors, such as GDP per capita and government health expenditure (as a share of GDP), influence health outcomes in SSA. Our analysis indicates that countries with higher GDP per capita tend to experience improved health outcomes. This observation is consistent with Grossman's [35, 36] framework, which suggests that increased income enables individuals to invest more in their health. Similarly, Gerring et al. [64] highlight that higher income provides the means to satisfy essential needs, including adequate nutrition, medical care and vaccinations, while also facilitating improved access to sanitation, education, housing and employment, factors that collectively contribute to better population health.

Similarly, higher government health expenditure (% of GDP) appears to be associated with increased LE at birth, although the effect is not statistically significant. The non‐significant effect on LE may reflect that spending is often targeted toward specific programs, such as maternal and child health interventions, rather than broad improvements in overall longevity [65]. Moreover, inefficiencies in health system governance may limit the impact of expenditures on LE, which aligns with the study's focus on how governance moderates health outcomes [26]. Nonetheless, such expenditure plays an important role in reducing under‐5 and maternal mortality rates in SSA, consistent with the evidence provided by Nketiah‐Amponsah [66]. This pattern likely reflects the fact that public investments in the health sector are often directed towards maternal and child health programs, preventive services and strengthening primary healthcare infrastructure. Although improvements in overall LE may take longer to manifest, the immediate effects are more visible in reductions in maternal and child mortality.

Access to primary school, safe drinking water, sanitation, rate of access to electricity and the proportion of the population living in urban areas constitute factors that can also explain health in SSA. We find that education is good for health in SSA. This result is consistent with the prediction of the Grossman model [35, 36]: education has positive and statistically significant coefficients in the health demand function. It is also consistent with other previous studies, such as Balaj et al. [67] and Chen et al. [19]. The authors revealed that educated people tend to avoid or reduce the risks associated with several unhealthy behaviours like smoking, excessive alcohol consumption and physical inactivity.

Although access to safe drinking water is not significant, it does help to improve health outcomes. One possible explanation is multicollinearity with other covariates, such as sanitation coverage and GDP per capita, which may absorb part of its effect [18]. Additionally, there may be measurement limitations in the WDI data for this indicator, particularly in some SSA countries, which could reduce precision [68].

The non‐significant effects of access to safe drinking water and government health expenditure on some health outcomes may reflect multicollinearity with other covariates, such as sanitation coverage and GDP per capita, which can absorb part of their explanatory power. Additionally, measurement limitations in cross‐country datasets in some SSA countries could reduce the precision of these estimates [18, 68]. These considerations provide context for the observed non‐significant coefficients and do not detract from the overall findings regarding the role of the skilled health workforce and governance.

Improving access to sanitation significantly contributes to reducing maternal mortality in SSA. This finding aligns with the WHO [8], which highlights that adequate household sanitation is strongly associated with better maternal health outcomes. These benefits include a reduced likelihood of pregnancy complications, fewer delivery‐related risks and improved neonatal survival and birth outcomes.

### 5.2 Robustness Check

To ensure that our results are not driven solely by the construction of the aggregate governance index, a robustness check is performed using government effectiveness from the WGI as an individual dimension. Government effectiveness is particularly relevant for health outcomes, as it captures the quality of public services, policy implementation and the capacity of the civil service to deliver essential health interventions [54]. Results (see Supporting Information Tables A1–A3) indicate that the positive moderating effect of governance on the relationship between skilled health workforce density and health outcomes remains consistent when using government effectiveness alone, confirming that our findings are robust to alternative specifications of governance. This approach aligns with prior studies emphasising the importance of examining governance dimensions separately when analysing health outcomes [69].

The study demonstrates a significant interaction between skilled health workforce density and overall governance quality, indicating that stronger governance enhances the effectiveness of health workforce investments on health outcomes. However, as an aggregate governance measure is used, averaging six governance dimensions, the study cannot explore how the effect of workforce density varies at specific governance levels or for individual governance components. Future research could examine marginal effects across low, medium and high governance contexts or investigate the role of particular governance dimensions (e.g., government effectiveness and corruption control) to provide more nuanced policy guidance.

## 6 Conclusion

The purpose of this study is to examine the relationship between skilled health workforce professionals, particularly doctors, nurses and midwives and health outcomes in SSA, with a particular focus on the role of governance. Using data from the WDI of the World Bank for 45 SSA countries over the period 1996–2023, we employed the two‐step GMM to address potential endogeneity concerns.

The findings in Tables 2, 3, 4 demonstrate that the skilled health workforce significantly improves health outcomes in SSA. Specifically, a 1% increase in the skilled health workforce is associated with a 0.034‐year increase in LE, a 0.044‐point reduction in the MMR, and a negative association with under‐5 mortality. The interaction term between the governance aggregate value and health workforce density is positive and statistically significant (*β* = 0.399), highlighting the moderating role of governance in enhancing the effectiveness of health professionals. GDP per capita and primary school enrolment are positively associated with LE, whereas urban population and government health expenditure (% of GDP) exhibit mixed significance. Access to safe drinking water and sanitation shows variable effects across different health outcomes.

The results indicate that the skilled health workforce has a positive impact on health outcomes in the region. Governance emerges as a critical moderating factor: Improvements in governance quality not only enhance the effectiveness of health professionals but also contribute directly to better population health. Additionally, countries with higher GDP per capita and greater government health expenditure (as a share of GDP) generally achieve more favourable health outcomes. Beyond economic and institutional factors, structural variables, such as access to primary education, safe drinking water, improved sanitation and urbanisation, also play important roles in shaping health outcomes in SSA.

To strengthen health outcomes, policymakers should focus on two complementary strategies. First, enhancing governance through transparent decision‐making, effective accountability mechanisms and robust regulatory frameworks can reduce corruption, limit resource leakages and create an enabling environment for health professionals to deliver quality care. Decentralising health management and promoting community participation in decision‐making are also vital for sustaining long‐term improvements in population health. Second, expanding the skilled health workforce is essential to meet SSA's growing healthcare needs. Governments should invest in training and continuous professional development, recruit and retain qualified doctors, nurses and midwives and provide competitive remuneration, especially in underserved rural areas where shortages are most acute. Complementary investments in education, safe water and sanitation will further reinforce these efforts and support sustained improvements in health outcomes across the region.

## Author Contributions

**Michael Kouadio**: conceptualisation, writing – original draft, methodology, writing – review and editing, software, formal analysis.

## Funding

The author has nothing to report.

## Ethics Statement

The author has nothing to report.

## Conflicts of Interest

The author declares no conflicts of interest.

## Supporting information




**Supporting File 1:** Table A1: Relationship between skilled health workforce, governance quality and life expectancy at birth in SSA. **Table A2:** Relationship between health workforce, governance quality and under‐5 mortality rate in SSA. **Table A3:** Relationship between skilled health workforce, governance quality and maternal mortality ratio in SSA.

## Data Availability

The data used in this study are publicly available from the World Bank's World Development Indicators (WDI) and the Worldwide Governance Indicators (WGI) databases. These datasets can be accessed at https://databank.worldbank.org/source/world-development-indicators and https://info.worldbank.org/governance/wgi/. All data used in this analysis are available upon reasonable request from the corresponding author.

## References

[1] United Nations . The Sustainable Development Goals Report 2020, (2020). https://unstats.un.org/sdgs/report/2020/.

[2] African Union , African Union Commission (African Union, 2011), https://au.int/en/commission.https://au.int/sites/default/files/bids/42353‐ClimSA_‐_Invitation_to_Bid‐_Supply_Delivery_Installation_of_Earth_Station_and_Training_edit_PK.pdf.

[3] J. Murunga , E. G. Mogeni , and D. N. Kimolo , “Public Health Spending and Health Outcomes in Kenya, ” European Scientific Journal ESJ 15 (2019): 125–138, http://ir.mksu.ac.ke/handle/123456780/4198.

[4] R. Barro , Health and Economic Growth (World Health Organization, 1996), 1–47.

[5] G. S. Becker , Human Capital: A Theoretical and Empirical Analysis, With Special Reference to Education (University of Chicago Press, 1964).

[6] C. Goldin , Human Capital, Inequality, and Economic Growth, (Use exact journal/book source as in your paper) (2016).

[7] WHO , Global Strategy on Human Resources for Health: Workforce 2030, (2020), https://www.who.int/publications/i/item/9789241511131. 10.1186/s12960-024-00940-x PMC11450981 39363378

[8] WHO , Nursing and Midwifery Personnel (Per 10 000 Population), (2022), https://www.who.int/data/gho/data/indicators/indicator‐details/GHO/nursing‐and‐midwifery‐personnel‐(per‐10‐000‐population).

[9] S. C. E. Anyangwe and C. Mtonga , “Inequities in the Global Health Workforce : The Greatest Impediment to Health in Sub‐Saharan Africa, ” International Journal of Environmental Research and Public Health 4, no. 2 (2007): 93–100, 10.3390/ijerph2007040002. 17617671 PMC3728573

[10] A. Hagopian , M. J. Thompson , M. Fordyce , K. E. Johnson , and L. G. Hart , “The Migration of Physicians From Sub‐Saharan Africa to the United States of America: Measures of the African Brain Drain,” Human Resources for Health 2 (2004): 17, 10.1186/1478-4491-2-17. 15598344 PMC544595

[11] World Health Organization Regional Office for Africa , Health Workforce in the WHO African Region: Challenges and Policy Options (2021), https://www.afro.who.int.

[12] A. Ahmat , S. C. Okoroafor , and J. Kazadi , Health Workforce Shortages and Projections in Africa (World Health Organization, 2022).

[13] G. Bamba , J. A. Abboud , E. Olal , and D. L. Kitara , “Comparative Analysis of the Evolution of Life Expectancy in the United Republic of Tanzania, Uganda, and Kenya in 61 Years (1960‐2021): A Secondary Data Analysis of the World Population Prospects (WPPs) on the Three East African Countries,” medRxiv (2024): 2024–2034.

[14] OECD Health at a Glance 2024: OECD Indicators (OECD Publishing, 2024), https://www.oecd.org/health/health‐at‐a‐glance/.

[15] R. Musarandega , M. Nyakura , R. Machekano , R. Pattinson , and S. P. Munjanja , “Causes of Maternal Mortality in Sub‐Saharan Africa: A Systematic Review of Studies Published From 2015 to 2020,” Journal of Global Health 11 (2021): 04048, 10.7189/jogh.11.04048. 34737857 PMC8542378

[16] D. Tiruneh , N. Assefa , and B. Mengiste , “Perinatal Mortality and Its Determinants in Sub Saharan African Countries: Systematic Review and Meta‐analysis,” Maternal Health, Neonatology and Perinatology 7, no. 1 (2021): 1, 10.1186/s40748-020-00120-4. 33386082 PMC7775631

[17] T. O. Popoola , “Immunisation and Infant Mortality in Sub‐Saharan African Countries, ” Ilorin Journal of Economic Policy 10, no. 1 (2023): 46–57.

[18] WHO , WASH Safely Managed Drinking‐Water Services, Population Using (%), (2025), https://www.who.int/data/gho/data/indicators/indicator‐details/GHO/population‐using‐safely‐managed‐drinking‐water‐services‐(‐).

[19] J. Chen , L. Wei , and F. Manzoor , “Bridging the Gap : How Education Transforms Health Outcomes and Influences Health Inequality in Rural china, ” Frontiers in Public Health 12 (2024): 1437630, 10.3389/fpubh.2024.1437630. 39540097 PMC11557493

[20] D. E. Bloom , D. Canning , and J. Sevilla , “The Effect of Health on Economic Growth: A Production Function Approach,” World Development 32, no. 1 (2004): 1–13, 10.1016/j.worlddev.2003.07.002.

[21] G. Dussault and M. C. Franceschini , “Not Enough There, Too Many Here: Understanding Geographical Imbalances in the Distribution of the Health Workforce,” Human Resources for Health 4 (2006): 12, 10.1186/1478-4491-4-12. 16729892 PMC1481612

[22] R. Mash , G. Ogunbanjo , and S. Naidoo , “Strengthening Health Systems in Africa: Primary Health Care and Workforce Development,” BMJ Global Health (2022), 10.1136/bmjgh-2021-007123.

[23] I. Kickbusch and D. Gleicher , Governance for Health in the 21st Century (World Health Organization, Regional Office for Europe, 2012), https://www.euro.who.int.

[24] M. Marmot , “The Health Gap: The Challenge of an Unequal World,” The Lancet 386, no. 10011 (2015): 2442–2444, 10.1016/S0140-6736(15)00150-6. 26364261

[25] M. Kouadio , “Effects of Governance Quality on Health Outcomes in West African Countries,” Discover Public Health 22, no. 1 (2025): 645, 10.1186/s12982-025-01014-6. 39719359

[26] M. Kouadio and A. Njong Mom , “Health Expenditure, Governance Quality, and Health Outcomes in West African Countries, ” International Journal of Health Planning and Management 40, no. 2 (2025): 427–441, 10.1002/hpm.3887. 39719359

[27] A. V. Glotko , A. G. Polyakova , M. Y. Kuznetsova , K. E. Kovalenko , R. A. Shichiyakh , and M. V. Melnik , “Main Trends of Government Regulation of Sectoral Digitalization,” Entrepreneurship and Sustainability Issues 7, no. 3 (2020): 2181–2195, 10.9770/jesi.2020.7.3(48).

[28] D. Waldo , The Study of Public Administration (Рипол Классик, 1955).

[29] L. L. Liang and A. J. Mirelman , “Why Do some Countries Spend More for Health? An Assessment of Sociopolitical Determinants and International Aid for Government Health Expenditures,” Social Science & Medicine 114 (2014): 161–168, 10.1016/j.socscimed.2014.05.044. 24929917

[30] G. C. Montes and P. C. Paschoal , “Corruption: What Are the Effects on Government Effectiveness? Empirical Evidence Considering Developed and Developing Countries,” Applied Economics Letters 23, no. 2 (2016): 146–150, 10.1080/13504851.2015.1058900.

[31] N. Nadpara and S. Samanta , “An Empirical Examination of the Effect of Corruption on Health outcomes,” in The College of New Jersey (2015), 1–26.

[32] A. B. A. Jabbar , K. M. Talha , V. Nambi , D. Abramov , and A. M. K. Minhas , “Primary Care Physician Density and Mortality in the United States, ” Journal of the National Medical Association 116, no. 5 (2024): 600–606, 10.1016/j.jnma.2024.10.001. 39455301

[33] R. L. Thomas , C. Millett , R. D. Sousa Soares , and T. Hone , “More Doctors, Better Health? A Generalised Synthetic Control Approach to Estimating Impacts of Increasing Doctors Under Brazil's Mais Medicos Programme, ” Social Science & Medicine 358 (2024): 117222, 10.1016/j.socscimed.2024.117222. 39181082 PMC7618175

[34] D. C. North , Institutions, Institutional Change and Economic Performance (Cambridge Univ Pr, 1990), 10.1017/CBO9780511808678.

[35] M. Grossman , “On the Concept of Health Capital and the Demand for Health, ” Journal of Political Economy 80, no. 2 (1972): 223–255, 10.1086/259880.

[36] M. Grossman , “The Human Capital Model, ” in Handbook of Health Economics (Elsevier, 2000), https://www.sciencedirect.com/science/article/pii/S1574006400801663.

[37] J. I. Rosser , K. Z. Aluri , A. Kempinsky , S. Richardson , and E. Bendavid , “The Effect of Healthcare Worker Density on Maternal Health Service Utilization in Sub‐Saharan Africa,” The American Journal of Tropical Medicine and Hygiene 106, no. 3 (2022): 939, 10.4269/ajtmh.21-0727. 35026729 PMC8922518

[38] M. P. Nguyen , T. Mirzoev , and T. M. Le , “Contribution of Health Workforce to Health Outcomes: Empirical Evidence From Vietnam, ” Human Resources for Health 14, no. 1 (2016): 68, 10.1186/s12960-016-0165-0. 27852268 PMC5112617

[39] S. Liang , J. Macinko , D. Yue , and Q. Meng , “The Impact of the Health Care Workforce on Under‐Five Mortality in Rural China, ” Human Resources for Health 17, no. 1 (2019): 21, 10.1186/s12960-019-0357-5. 30885196 PMC6423838

[40] R. Nakatani , Q. Zhang , and G. Valdes , “Health Expenditure Decentralization and Health Outcomes : The Importance of Governance, ” Publius: The Journal of Federalism 54, no. 1 (2024): 59–87.

[41] D. K. Ciccone , T. Vian , L. Maurer , and E. H. Bradley , “Linking Governance Mechanisms to Health Outcomes: A Review of the Literature in Low‐and Middle‐Income Countries, ” Social Science & Medicine 117 (2014): 86–95. 25054281 10.1016/j.socscimed.2014.07.010

[42] K. Khan , M. Zeeshan , A. Moiz , et al., “Influence of Government Effectiveness, Health Expenditure, and Sustainable Development Goals on Life Expectancy: Evidence From Time Series Data, ” Sustainability 16, no. 14 (2024): 6128, 10.3390/su16146128.</bib.

[43] A. Hadipour , S. Delavari , and M. Bayati , “What Is the Role of Institutional Quality in Health Outcomes? A Panel Data Analysis on 158 Countries From 2001–2020, ” Heliyon 9, no. 9 (2023): e20251, 10.1016/j.heliyon.2023.e20251. 37809989 PMC10560016

[44] J. M. Kelley , G. Kraft‐Todd , L. Schapira , J. Kossowsky , and H. Riess , “The Influence of the Patient‐Clinician Relationship on Healthcare Outcomes: A Systematic Review and Meta‐Analysis of Randomized Controlled Trials,” PLoS ONE 9, no. 4 (2014): e94207, 10.1371/journal.pone.0094207. 24718585 PMC3981763

[45] M. S. Gupta , M. E. Tiongson , and M. Verhoeven , Does Higher Government Spending Buy Better Results in Education and Health Care? (International Monetary Fund, 1999).

[46] L. E. Pandolfelli , J. Shandra , and J. Tyagi , “The international monetary fund, Structural Adjustment, and Women's Health: A Cross‐national Analysis of Maternal Mortality in Sub‐Saharan Africa,” The Sociological Quarterly 55, no. 1 (2014): 119–142, 10.1111/tsq.12046.

[47] R. Gaitonde , A. D. Oxman , P. O. Okebukola , and G. Rada , “Interventions to Reduce Corruption in the Health Sector,” Cochrane Database of Systematic Reviews 2016, no. 8 (2016): CD008856, 10.1002/14651858.CD008856.pub2. 27528494 PMC5014759

[48] R. B. Saltman and R. Busse , Regulating Entrepreneurial Behaviour in European Health Care Systems, ed. Mossialos, E. , (Open University Press, 2002), 1–256.

[49] M. V. Achim , V. L. Văidean , and S. N. Borlea , “Corruption and Health Outcomes Within an Economic and Cultural Framework: MV Achim et al,” The European Journal of Health Economics 21, no. 2 (2020): 195–207, 10.1007/s10198-019-01120-8. 31587123

[50] A. L. Mohammed , I. N. Yakubu , and I. Abdallah , “The Interactive Effect of Control of Corruption and Government Health Expenditure on Health outcomes,” in Examining Corruption and the Sustainable Development Goals (IGI Global Scientific Publishing, 2024), 266–278.

[51] Y. Ding , L. Chin , F. Li , and P. Deng , “How Does Government Efficiency Affect Health Outcomes? The Empirical Evidence From 156 Countries,” International Journal of Environmental Research and Public Health 19, no. 15 (2022): 9436, 10.3390/ijerph19159436. 35954795 PMC9368218

[52] I. Ouedraogo , I. Dianda , and I. T. Adeyele , “Institutional Quality and Health Outcomes in Sub‐Saharan Africa, ” Research in Applied Economics 12, no. 4 (2020): 22–45.

[53] H. Adair‐Rohani , K. Zukor , and S. Bonjour , “Limited Electricity Access in Health Facilities of sub‐Saharan Africa: A Systematic Review of Data on Electricity Access, Sources, and Reliability,” Global Health: Science and Practice 1, no. 2 (2013): 249–261. 25276537 10.9745/GHSP-D-13-00037 PMC4168575

[54] D. Kaufmann , A. Kraay , and M. Mastruzzi , “The Worldwide Governance Indicators: Methodology and Analytical Issues, ” Hague Journal on the Rule of Law 3, no. 2 (2011): 220–246.

[55] S. Holmberg and B. Rothstein , “Dying of Corruption. *Health Economics* ,” Policy and Law 6, no. 4 (2011): 529–547. 10.1017/S174413311000023X 20809992

[56] R. J. Little and D. B. Rubin , Statistical Analysis With Missing Data (John Wiley & Sons, 2019), https://books.google.com/books?hl=en&lr=&id=BemMDwAAQBAJ&oi=fnd&pg=PR11&dq=Statistical+analysis+with+missing+data&ots=FCFR8ZGYZZ&sig=N3CIXU0pC‐Tuy‐9lxrzd1iq5LzE.

[57] M. Arellano and S. Bond , “Some Tests of Specification for Panel Data: Monte Carlo Evidence and an Application to Employment Equations,” The Review of Economic Studies 58, no. 2 (1991): 277–297, 10.2307/2297968.

[58] S. Ullah , P. Akhtar , and G. Zaefarian , “Dealing With Endogeneity Bias: The Generalized Method of Moments (GMM) for Panel Data,” Industrial Marketing Management 71 (2018): 69–78, 10.1016/j.indmarman.2017.11.010.

[59] D. Roodman , “How to Do xtabond2: An Introduction to Difference and System GMM in Stata,” The Stata Journal 9, no. 1 (2009): 86–136, 10.1177/1536867X0900900106.

[60] S. R. Bond , “Dynamic Panel Data Models : A Guide to Micro Data Methods and Practice, ” Portuguese Economic Journal 1, no. 2 (2002): 141–162, 10.1007/s10258-002-0009-9.

[61] J. Liu and K. Eggleston , “The Association Between Health Workforce and Health Outcomes : A Cross‐Country Econometric Study, ” Social Indicators Research 163, no. 2 (2022): 609–632, 10.1007/s11205-022-02910-z. 35310535 PMC8919693

[62] M. E. Kruk , L. P. Freedman , G. A. Anglin , and R. J. Waldman , “Rebuilding Health Systems to Improve Health and Promote Statebuilding in Post‐Conflict Countries: A Theoretical Framework and Research Agenda,” Social Science & Medicine 70, no. 1 (2010): 89–97, 10.1016/j.socscimed.2009.09.042. 19850390

[63] M. Afzal , G. Cometto , E. Rosskam , and M. Sheikh , “Global Health Workforce Alliance : Increasing the Momentum for Health Workforce Development, ” Revista Peruana De Medicina Experimental Y Salud Publica 28, no. 2 (2011): 298–307. 21845311

[64] J. Gerring , S. C. Thacker , and R. Alfaro , “Democracy and Human Development,” The Journal of Politics 74, no. 1 (2012): 1–17, 10.1017/S0022381611001113.

[65] W. N. S. Kabongo and J. Mbonigaba , “Public Health Spending in Sub‐Saharan Africa : Exploring Transmission Mechanisms Using the Latent Growth Curve Mediation Model, ” Health Economics Review 14 (2024): 14, 10.1186/s13561-023-00472-5. 38372932 PMC10875913

[66] E. Nketiah‐Amponsah , “The Impact of Health Expenditures on Health Outcomes in Sub‐Saharan Africa,” Journal of Developing Societies 35, no. 1 (2019): 134–152, 10.1177/0169796X19826759.

[67] M. Balaj , C. A. Henson , A. Aronsson , et al., “Effects of Education on Adult Mortality : A Global Systematic Review and Meta‐Analysis, ” Lancet Public Health 9, no. 3 (2024): e155–e165. 38278172 10.1016/S2468-2667(23)00306-7 PMC10901745

[68] World Bank , World Development Indicators | DataBank (World Bank, 2025), https://databank.worldbank.org/source/world‐development‐indicators.

[69] S. Gupta , H. Davoodi , and E. Tiongson , “Corruption and the Provision of Health Care and Education services, ” in The Political Economy of Corruption (Routledge, 2001).

