# Supplementary material for: Better Governance and Skilled Health Workforce Density: Are They Twin Catalysts for Health Outcomes in Sub‐Saharan Africa?
Source: Public Health Chall. 2026 Apr 21;5(2):e70235. doi: 10.1002/puh2.70235 (PMC13098659; doi:10.1002/puh2.70235)
Supplement: Supplementary file 1 — Supporting File 1: Table A1: Relationship between skilled health workforce, governance quality and life expectancy at birth in SSA. Table A2: Relationship between health workforce, governance quality and under‐5 mortality rate in SSA. Table A3: Relationship between skilled health workforce, governance quality and maternal mortality ratio in SSA. [file PUH2-5-e70235-s001.docx]

Appendices

Table A1. Relationship between skilled health workforce, Governance Quality, and Life Expectancy at Birth in SSA.

| Life Expectancy at Birth | | | | | |  |
| --- | --- | --- | --- | --- | --- | --- |
| Variable | Coef. | St.Err. | t-value | p-value | [95% Conf. Interval] | Sig |
| Life Expectancy at Birth (Lag 1) | 0.810 | 0.006 | 134.84 | 0.000 | 0.799 0.822 | *** |
| Health workforce | 0.753 | 0.140 | 5.38 | 0.000 | 0.479 1.027 | *** |
| Government effectiveness × Skilled Health Workforce | 0.842 | 0.182 | 4.63 | 0.000 | 0.486 1.198 | *** |
| Government effectiveness | 0.329 | 0.053 | 6.15 | 0.000 | 0.224 0.433 | *** |
| Urban population | 0.010 | 0.003 | 3.76 | 0.000 | 0.005 0.015 | *** |
| Sanitation | 0.012 | 0.002 | 5.49 | 0.000 | 0.008 0.017 | *** |
| Access to basic drinking water services | 0.006 | 0.003 | 1.85 | 0.065 | 0.000 0.012 | * |
| Ln GDP per capita | 0.071 | 0.032 | 2.25 | 0.024 | 0.009 0.133 | ** |
| Government health expenditure (%GDP) | 0.002 | 0.002 | 0.99 | 0.322 | -0.005 0.002 |  |
| Primary school enrolment | 0.007 | 0.001 | 12.79 | 0.000 | 0.006 0.008 | *** |
| Constant | 9.669 | 0.176 | 54.84 | 0.000 | 9.323 10.014 | *** |
|  |  |  |  |  |  |  |
| Mean dependent var 8.302 SD dependent var 7.356 | | | | | | |
|  | | | | | | |
| Number of observations 1215 Number of instruments: 16 | | | | | | |
| Number of groups 45 | | | | | |  |
| AR(1) 0.000 | | | | | |  |
| AR(2) 0.321 | | | | | |  |
| Sargan test | | | | | | 0.000 |
| Hansen test | | | | | | 0.899 |
|  |  |  |  |  |  |  |

| Source: Author’s compilation, WDI data (1996-2023); *Significance level *** p<.01, ** p<.05, * p<.1* |
| --- |

**Table A2: Relationship between health workforce, Governance Quality, and Under-5 Mortality Rate in SSA**

| Under-5 Mortality Rate | |  |  |  |  |  |
| --- | --- | --- | --- | --- | --- | --- |
| Variable | Coef. | St.Err. | t-value | p-value | [95% Conf. Interval] | Sig |
| Under-5 Mortality Rate (lag 1) | 0.797 | 0.002 | 423.82 | 0.000 | 0.793 0.801 | *** |
| Skilled Health Workforce Density | 0.430 | 0.584 | 0.74 | 0.461 | -0.714 1.574 |  |
| Governance effectiveness× Skilled Health Workforce | -0.587 | 0.435 | -1.35 | 0.013 | -1.002 -0.172 | ** |
| Governance Effectiveness | -0.923 | 0.082 | -11.29 | 0.000 | -1.084 -0.763 | *** |
| Urban Population | -0.022 | 0.006 | -3.68 | 0.000 | -0.034 -0.011 | *** |
| Sanitation | -0.060 | 0.010 | -5.82 | 0.000 | -0.080 -0.040 | *** |
| Access to Basic Drinking Water Services | -0.010 | 0.007 | -1.46 | 0.144 | -0.024 0.003 |  |
| Ln GDP per Capita (lngdppc) | -0.487 | 0.082 | -5.95 | 0.000 | -0.647 -0.327 | *** |
| Government Health Expenditure (%GDP) | -0.037 | 0.004 | -9.45 | 0.000 | -0.045 -0.030 | *** |
| Primary School Enrollment | -0.022 | 0.002 | -14.23 | 0.000 | -0.025 -0.019 | *** |
| Constant | 18.593 | 0.734 | 25.33 | 0.000 | 17.155 20.032 | *** |
|  |  |  |  |  |  |  |
| Mean dependent var: 59.330  F-test of Joint Significance 0.0000  Number of observations: 1215  Number of instruments: 18  Number of groups (countries): 45  AR(1) test: 0.019  AR(2) test: 0.238  Sargan test: 0.000  Hansen test: 1.000 | | | |  |  |  |

| Source: Author’s compilation, WDI data (1996-2023); *Significance level *** p<.01, ** p<.05, * p<.1* |
| --- |

Table A3: Relationship between skilled health workforce, Governance Quality, and Maternal Mortality Ratio in SSA

| Variable | Coef. | St.Err. | t-value | p-value | [95% Conf. Interval] | Sig |
| --- | --- | --- | --- | --- | --- | --- |
| Maternal Mortality Rate (Lag 1) | 0.927 | 0.013 | 69.03 | 0.000 | 0.900 0.953 | *** |
| Skilled Health Workforce Density | -0.085 | 0.013 | -6.55 | 0.000 | -0.110 -0.059 | *** |
| Governance effectiveness × Skilled Health Workforce | -0.109 | 0.037 | -2.96 | 0.003 | -0.181 -0.037 | *** |
| Governance Effectiveness | -0.029 | 0.008 | -3.44 | 0.001 | -0.045 -0.012 | *** |
| Urban Population | -0.022 | 0.006 | -3.68 | 0.000 | -0.034 -0.011 | *** |
| Sanitation | -0.001 | 0.000 | -2.18 | 0.029 | -0.003 -0.001 | ** |
| Access to Basic Drinking Water Services | -0.010 | 0.007 | -1.46 | 0.144 | -0.024 0.003 |  |
| Ln GDP per capita | -0.487 | 0.082 | -5.95 | 0.000 | -0.647 -0.327 | *** |
| Government Health Expenditure (%GDP) | -0.001 | 0.000 | -3.43 | 0.001 | -0.002 - 0.001 | *** |
| Primary School Enrollment | -0.022 | 0.002 | -14.23 | 0.000 | -0.025 -0.019 | *** |
| Constant | 0.467 | 0.098 | 4.77 | 0.000 | 0.275 0.658 | *** |

Mean dependent var: 59.330

F-test of Joint Significance (0.0000)

Number of observations: 1215

Number of instruments: 18

Number of groups (countries): 45

AR(1) test: 0.044

AR(2) test: 0.313

Sargan test: 0.000

Hansen test: 1.000

| Source: Author’s compilation, WDI data (1996-2023); *Significance level *** p<.01, ** p<.05, * p<.1* |
| --- |
